# Supplementary material for: HDAC6 Negatively Regulates miR-155-5p Expression to Elicit Proliferation by Targeting RHEB in Microvascular Endothelial Cells under Mechanical Unloading
Source: Int J Mol Sci. 2021 Sep 29;22(19):10527. doi: 10.3390/ijms221910527 (PMC8508889; doi:10.3390/ijms221910527)
Supplement: Supplementary file 1 [file ijms-22-10527-s001.zip › ijms-1370275-SI.pdf]

Supplementary Materials for

HDAC6 negatively regulates miR-155-5p expression to elicit proliferation by targeting RHEB in microvascular endothelial cells under mechanical unloading

Liqun Xu[1+], Lijun Zhang[1+], Xiaoyan Zhang[1], Gaozhi Li[1], Yixuan Wang[1], Jingjing Dong[1], Honghui Wang[1,2], Zebing Hu[1], Xinsheng Cao[1], Shu Zhang[1*], Fei Shi[1*]

[*]To whom correspondence should be addressed: Shu Zhang at shuzhang89@hotmail.com; Fei Shi at shifei719@fmmu.edu.cn.

Table S1 Primer sequences used for RT-qPCR

Gene	Sequence（5'~3'）
U6-F	CTCGCTTCGGCAGCACA
U6-R	AACGCTTCACGAATTTGCGT
mmu-miR-155-5p	CCGCGTTAATGCTAATTGTGATAGGGG
mmu-miR-375-3p	TTTGTTCGTTCGGCTCGCGT
mmu-miR-7a-5p	CGCGTGGAAGACTAGTGATTTTGTTGT
mmu-miR-212-3p	TAACAGTCTCCAGTCACGGCC
mmu-miR-206-3p	CCGTGGAATGTAAGGAAGTGTGTGG
mmu-miR-532-5p	CATGCCTTGAGTGTAGGACCGT
mmu-miR-181d-5p	CCAACATTCATTGTTGTCGGTGGGT
mmu-miR-183-5p	CCGCTATGGCACTGGTAGAATTCACT
mRQ 3' Primer	TGGTGTCGTGGAGTCG
GAPDH-F	TGTGTCCGTCGTGGATCTGA
GAPDH-R	TTGCTGTTGAAGTCGCAGGAG
RHEB-F	AAGAAGGACCTGCATATGGAAA
RHEB-R	GAAGACTTTCCTTGTGAAGCTG
HDAC6-F	AGAAGATGATGAGTCACTGCAA
HDAC6-R	GTGACACATGATGCGTAAGATG

Table S2 RNA oligo sequences for transfection

Name	Sequence (5' - 3')
**Mimic-NC sense**	UUCUCCGAACGUGUCACGUTT
**Mimic-NC antisense**	ACGUGACACGUUCGGAGAATT
**Inhibitor-NC**	CAGUACUUUUGUGUAGUACAA
**Mimic-155-5p sense**	UUAAUGCUAAUUGUGAUAGGGGU
**Mimic-155-5p antisense**	CCCUAUCACAAUUAGCAUUAAUU
**Inhibitor-155-5p**	ACCCCUAUCACAAUUAGCAUUAA
**Negative control sense**	UUCUCCGAACGUGUCACGUTT
**Negative control antisense**	ACGUGACACGUUCGGAGAATT
**siRNA-HDAC6 sense**	CCCAUCGCCUAUGAGUUUATT
**siRNA-HDAC6 antisense**	UAAACUCAUAGGCGAUGGGTT
**siRNA-RHEB sense**	GCCAAUUUGUUGAUUCCUATT
**siRNA-RHEB antisense**	UAGGAAUCAACAAAUUGGCTT

Figure S1:

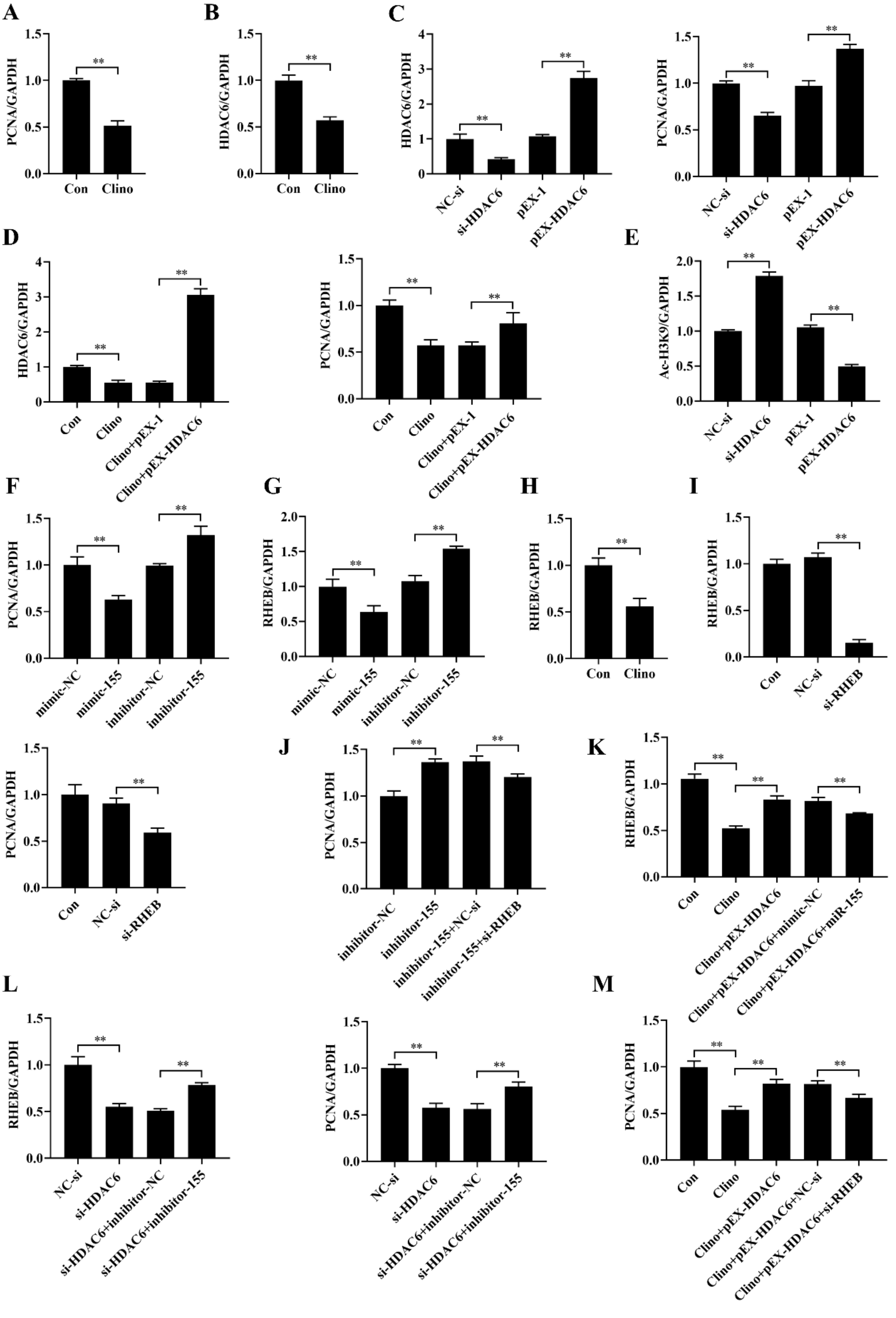

(A) Protein expression of PCNA was analyzed by Western blotting in microvascular endothelial cells that were cultured under clinorotation conditions for 48 h (N = 3). (B) The HDAC6 protein

level was analyzed by Western blotting (N = 3); (C) The HDAC6 and PCNA protein levels in microvascular endothelial cells were analyzed by Western blotting after transfection with HDAC6 siRNA or pEX-HDAC6 (N = 3); (D) The protein expression of HDAC6 and PCNA was analyzed by Western blotting after microvascular endothelial cells were transfected with pEX-HDAC6 and then cultured under mechanical unloading conditions for 48 h (N = 3); (E) Ac-H3K9 protein expression was analyzed by Western blotting in microvascular endothelial cells treated with HDAC6 siRNA, pEX-HDAC6 or the corresponding controls for 48 h (N = 3); (F) The protein expression of PCNA in microvascular endothelial cells was analyzed by Western blotting (N = 3); (G) Western blotting analysis of the protein expression of RHEB (N = 3); (H) The RHEB protein level was analyzed by Western blotting (N = 3); (I) The RHEB and PCNA protein levels in microvascular endothelial cells were analyzed by Western blotting after transfection with RHEB siRNA (N = 3); (J) PCNA protein levels were measured by Western blotting after co-transfecting inhibitor-155-5p and siRNA-RHEB into microvascular endothelial cells (N = 3); (K) RHEB protein expression was analyzed by Western blotting after microvascular endothelial cells were transfected with pEX-HDAC6 alone or pEX-HDAC6 plus mimic-155 and subjected to clinorotation unloading for 48 h (N = 3); (L) Western blotting analysis of RHEB and PCNA protein expression (N = 3); (M) PCNA protein expression was analyzed by Western blotting after microvascular endothelial cells were transfected with pEX-HDAC6 alone or pEX-HDAC6 plus siRNA-RHEB and subjected to clinorotation unloading for 48 h (N = 3).
